# Dynamics of Phloridzin and Related Compounds in Four Cultivars of Apple Trees during the Vegetation Period

**DOI:** 10.3390/molecules26133816

**Published:** 2021-06-22

**Authors:** Jan Táborský, Josef Sus, Jaromír Lachman, Barbora Šebková, Anežka Adamcová, Dalibor Šatínský

**Affiliations:** 1 Department of Chemistry, Faculty of Agrobiology, Food and Natural Resources, Czech University of Life Sciences Prague, 165 00 Prague, Czech Republic; taborsky@af.czu.cz (J.T.); lachman@af.czu.cz (J.L.); sebina07@seznam.cz (B.Š.); 2 Department of Horticulture, Faculty of Agrobiology, Food and Natural Resources, Czech University of Life Sciences Prague, 165 00 Prague, Czech Republic; sus@af.czu.cz; 3 Department of Analytical Chemistry, Faculty of Pharmacy in Hradec Králové, Charles University, 500 05 Hradec Králové, Czech Republic; adamcoa1@faf.cuni.cz

**Keywords:** phloridzin, phenolic compounds, bark and twigs, leaves and buds, apple tree cultivars, vegetation period

## Abstract

Apple trees (*Malus domestica* Borgh) are a rich source of dihydrochalcones, phenolic acids and flavonoids. Considering the increasing demand for these phytochemicals with health-benefitting properties, the objective of this study was to evaluate the profile of the main bioactive compounds—phloridzin, phloretin, chlorogenic acid and rutin—in apple tree bark, leaves, flower buds and twigs. The variety in the phenolic profiles of four apple tree cultivars was monitored during the vegetation period from March to September using chromatography analysis. Phloridzin, the major glycoside of interest, reached the highest values in the bark of all the tested cultivars in May (up to 91.7 ± 4.4 mg g^−1^ of the dried weight (DW), cv. ‘Opal’). In the leaves, the highest levels of phloridzin were found in cv. ‘Opal’ in May (82.5 ± 22.0 mg g^−1^ of DW); in twigs, the highest levels were found in cv. ‘Rozela’ in September (52.4 ± 12.1 mg g^−1^ of DW). In the flower buds, the content of phloridzin was similar to that in the twigs. Aglycone phloretin was found only in the leaves in relatively low concentrations (max. value 2.8 ± 1.4 mg g^−1^ of DW). The highest values of rutin were found in the leaves of all the tested cultivars (10.5 ± 2.9 mg g^−1^ of DW, cv. ‘Opal’ in September); the concentrations in the bark and twigs were much lower. The highest content of chlorogenic acid was found in flower buds (3.3 ± 1.0 mg g^−1^ of DW, cv. ‘Rozela’). Whole apple fruits harvested in September were rich in chlorogenic acid and phloridzin. The statistical evaluation by Scheffe’s test confirmed the significant difference of cv. ‘Rozela’ from the other tested cultivars. In conclusion, apple tree bark, twigs, and leaves were found to be important renewable resources of bioactive phenolics, especially phloridzin and rutin. The simple availability of waste plant material can therefore be used as a rich source of phenolic compounds for cosmetics, nutraceuticals, and food supplement preparation.

## 1. Introduction

Apple consumption is associated with beneficial health properties due to their high amount of phenolic compounds. However, there exists only limited information about their content in the individual organs of apple trees. Therefore, we discuss in this study the content of phloridzin and the other main phenolic compounds found in apple leaves, bark, buds and twigs.

**Figure 1:**
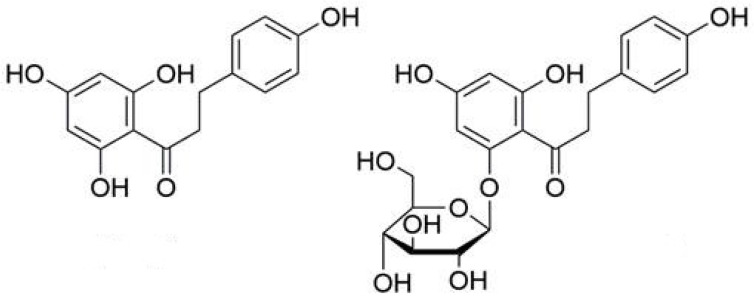
Chemical structure of phloretin and phloridzin.

Phloridzin (phloretin-2′-β-d-glucopyranoside, Figure 1), a plant glycoside belonging to the class of dihydrochalcones, represents one of the most significant bioactive compounds occurring in apple trees (*Malus* spp.). In last two decades, especially, phloridzin (sometimes called phlorizin) and its aglycone phloretin (Figure 1) were referred to many times for their beneficial effects for human health and various biological activities studied in animals.

Most importantly, phloridzin and its aglycone phloretin have a significant ability to reduce blood glucose levels [1,2,3,4,5]. Comprehensive reviews of natural phenolics as leading compounds for sodium glucose cotransporter inhibitors (SGLT) have been published [6,7]. Ehrenkranz et al. described the clinical pharmacology and toxicology of phloridzin, including investigational uses of phloridzin and its analogues in the treatment of diabetes, obesity and stress hyperglycemia [8].

Phloridzin can be utilized as a penetration enhancer of administered drugs because of its ability to bind to biological membranes and increase their fluidity [9]. Phloridzin and its derivatives show strong antioxidant [10,11,12,13], antimicrobial [14] and anti-inflammatory activities [15,16,17], including significant protection against cardiovascular [18] and liver diseases [19]. Another important effect of phloridzin is its association with protective effects against tumor growth, triggering cell death in malignant cells by either direct or indirect mechanisms [20]. However, in another study assessment of the in vitro cytotoxicity of extracts from the leaves of *Malus domestica* against human cancer, this was not confirmed, although their antioxidant, antimicrobial and lymphocyte proliferation were found [16]. The comparative analysis of dihydrochalcones’ antioxidant capacities assessed the pairs in the following relationship: phloretin > phloridzin, phloretin > trilobatin, trilobatin > phloridzin, indicating the maximum antioxidant capacity for aglycone phloretin [21]. Thus, glycosylation hinders the ability of radical adduct formation and reduces electron transfer and hydrogen atom transfer. In a recent study, Yin et al. also reported the coagulant activities of phloridzin and phloretin, among other flavonoid compounds such as glycosides of kaempferol in *Malus pumila* flowers [22]. Liu et al. reported the preparation of a natural yellow pigment from phloridzin (using enzymatic oxidation by tyrosinase), which can favourably contribute to the colour of apple juices and ciders [23].

According to the results published up to now, the most significant source of phloridzin and its analogues (sieboldin, trilobatin) is the apple tree (*Malus* spp.). Many reports have been devoted to the biosynthesis, occurrence, and possibilities of extraction of these compounds. A key study about the biosynthesis of phloridzin in apple trees was published by Gosch et al., describing three principal steps of biosynthesis via *p*-coumaroyl-CoA [24]. The comparative investigations between apple and pear trees were performed on young leaves of *M. domestica* cv. ‘Rebella’ and *Pyrus communis* cv. ‘Abbé Fétel’ (grown in Germany), including the isolation and characterization of all of the key enzymes, and confirming the absence of phloridzin in closely related *P. communis*. The genome-wide identification of glycosyltransferases converting phloretin to phloridzin in *Malus* species was studied by Zhou et al. [25]. A new comprehensive review describing the biosynthetic pathways and metabolic engineering of plant dihydrochalcones in apple trees was published by Ibdah et al. [26].

Aside from of apple trees, the occurrence of phloridzin was described in about thirty plant species, e.g., in *Fragaria* × *ananassa* (Rosaceae), *Fagopyrum esculentum* (Polygonaceae) and *Vaccinium macrocarpon* (Ericaceae). However, in species other than *M. domestica*, only exceptionally low amounts of phloridzin were detected [27]. The dietary intake of phloridzin from natural occurrences in foods was evaluated by Niederberger et al. [28]. European people consume, on average, 0.7–7.5 mg/d of phloridzin; however, high-level consumers may eat up to 52 mg/d of phloridzin [28].

**Figure 2:**
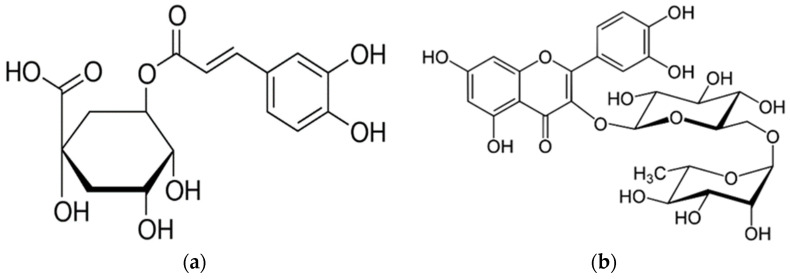
Chemical structure of chlorogenic acid (**a**) and rutin (**b**).

Chlorogenic acid (Figure 2) is a phenolic compound which is very frequent in the plant kingdom, functioning as an intermediate in lignin biosynthesis [29]. It is as an ester of caffeic and quinic acid. It is present in greater amounts mainly in coffee beans, and its protective effects on human health were discussed thoroughly in a comprehensive review by Tajik et al. [30]. Beside its other activities, chlorogenic acid shows significant antibacterial [31], antihypertensive [32], anti-obesity [33] and antioxidant properties [34]. The contents of chlorogenic acid in apple fruits were investigated many times in various connections [35,36,37]. However, only a small number of studies have been devoted to chlorogenic acid in apple leaves [38].

Rutin (Figure 2) is one of the most important flavonoid glycosides, consisting of aglycone quercetin and disaccharide rutinose (α-l-rhamnopyranosyl-(1→6)-β-d-glucopyranose). The most significant sources of rutin are buckwheat [39], apple fruits [40], citrus fruits [41] and green tea [42]. Its known effects on human health are the prevention and treatment of post-thrombotic syndrome [43]. The pharmacological potential of rutin was widely reported in a comprehensive review from Ganeshpurkar and Saluja summarizing the best current knowledge in this area [44].

It is known that apple consumption is associated with health benefits because apples are a rich source of phenolic compounds. The phenolic composition of apple fruits has been thoroughly investigated [35,36,37,40,45,46,47,48]. However, there exist only a few studies on the contents of these compounds in the individual organs of apple trees [49,50,51]. These studies indicate significantly higher levels of phenolic compounds. Moreover, a change in the content of these compounds during the growing season has not yet been investigated. Therefore, we focused in the present study on the following objectives. The main aims were: (i) to evaluate the levels of phloridzin, phloretin, chlorogenic acid and rutin in the twigs, bark, flower buds, and leaves of four apple tree cultivars grown in the Czech Republic; and (ii) to investigate their biochemical accumulation during the vegetation period of March–September.

## 2. Results and Discussion

### 2.1. Variations of Phloridzin, Phloretin, Chlorogenic Acid and Rutin in the Individual Parts of Apple Tree Cultivars during the Vegetation Period

**Figure 3:**
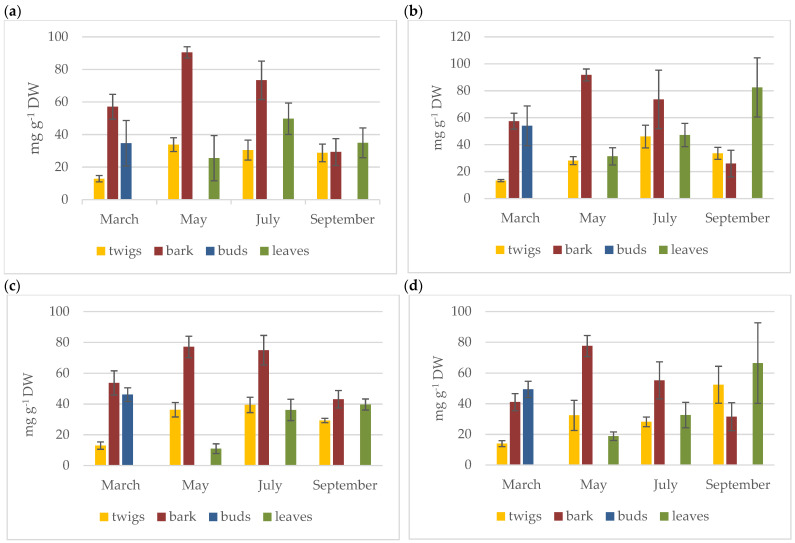
Content of phloridzin in (**a**) cv. ‘Jonagold’, (**b**) cv. ‘Opal’, (**c**) cv. ‘Redlane’, (**d**) cv. ‘Rozela’ during the vegetation period (*n* = 5).

The content of phloridzin and some related compounds (phloretin, chlorogenic acid, and rutin) in the individual parts of the investigated cultivars (twigs, bark, flower buds, and leaves) during the vegetation period are reported in the Supplementary Materials, Section 2, Tables S2–S5. The values of phloridzin (the principal compound of our interest) are depicted in Figure 3. All results are expressed in mg g^−1^ of dried weight (DW).

The highest levels of phloridzin were found in the bark of all the tested cultivars in the May period (cv. ‘Jonagold’ 90.5 mg g^−1^, cv. ‘Opal’ 91.7 mg g^−1^, cv. ‘Redlane’ 77.1 mg g^−1^, and cv. ‘Rozela’ 77.6 mg g^−1^). The contents of phloridzin in the leaves were highest in the period July–September (cv. ‘Jonagold’ 49.7 mg g^−1^ in July, cv. ‘Opal’ 82.5 mg g^−1^, ‘Redlane’ 39.7 mg g^−1^, and ‘Rozela’ 66.5 mg g^−1^ in September). In twigs, the maximum values of phloridzin varied in the period May–September (cv. ‘Jonagold’ 33.8 mg g^−1^ in May, cv. ‘Opal’ 46.0 mg g^−1^ in July, cv. ‘Redlane’ 39.4 mg g^−1^ in July, and cv. ‘Rozela’ 52.4 mg g^−1^ in September). The levels of phloridzin in the flower buds in March were 34.6 mg g^−1^ (cv. ‘Jonagold’), 53.9 mg g^−1^ (cv. ‘Opal’), 46.1 mg g^−1^ (cv. ‘Redlane’) and 49.4 mg g^−1^ (cv. ‘Rozela’). Significant contents of aglycone phloretin were found only in the leaves (maximum values of cv. ‘Jonagold’ 2.8 mg g^−1^ in May, cv. ‘Opal’ 2.0 mg g^−1^ in May, cv. ‘Redlane’ 1.3 mg g^−1^ in July, and cv. ‘Rozela’ 1.1 mg g^−1^ in May), while phloretin was not detected in the bark and twigs.

The estimation of two dihydrochalcones (phloridzin and phloretin) in four tested cultivars was completed, along with the determination of two other important phenolic compounds: chlorogenic acid and flavonol glycoside rutin. The highest levels of chlorogenic acid were found in flower buds in March (cv. ‘Jonagold’ 2.6 mg g^−1^, cv. ‘Opal’ 2.9 mg g^−1^, cv. ‘Redlane’ 2.6 mg g^−1^, and cv. ‘Rozela’ 3.3 mg g^−1^). The leaves were rich in rutin, especially in July and September (cv. ‘Jonagold’ 7.3 mg g^−1^ in July, cvs. ‘Opal’ 10.5 mg g^−1^, ‘Redlane’ 9.2 mg g^−1^, and ‘Rozela’ 8.5 mg g^−1^ in September).

Regarding the previous studies in this area, Rana et al. analysed apple leaves of *Malus domestica* Borkh. for their phenolic constituents and in vitro biological activity (cv. ‘Red Chief’, India) [16]. The average contents of phloridzin and phloretin in the leaves were 24.4 mg g^−1^ DW and 0.15 mg g^−1^ DW, respectively. The comparison with the maximum values of phloridzin and phloretin in the leaves in our study revealed their higher content in all four examined cultivars. An earlier analysis of the apple leaves of two cultivars grown in India [11] indicated average values of phloridzin of 52.0 mg g^−1^ DW for cv. ‘Golden’ and 20.3 mg g^−1^ DW for cv. ‘Royal’. These data correspond approximately with the data in our study, but cvs. ‘Opal’ and ‘Rozela’ achieved significantly higher phloridzin contents. The phloretin content in the leaves in the present study ranged from 1.1 mg g^−1^ to 2.8 mg g^−1^ DW and was found to be higher than 0.15 mg g^−1^ DW in the study by Rana et al. [16].

In the present study, phloretin was not detected in the bark, in which the highest amounts were characteristic for phloridzin, indicating that, under more gentle conditions of extraction, the glycoside phloridzin was not hydrolyzed to aglycone phloretin.

Parvaneh et al. determined the total phenolic, anthocyanin and flavonoid content, including rutin, chlorogenic acid, and many other phenolic compounds, in the leaves of three apple tree cultivars (‘Bastam’, ‘Bekran’ and ‘Red Delicious’) on three rootstocks (‘Bekran’, B9, and M9) [38]. ‘Bastam’ contained total phenolics of 93.6, ‘Bekran’ contained 117.8 and ‘Red Delicious’ contained 101.1 mg gallic acid/g DW; a total anthocyanin of 4.97, 4.22 and 3.61 mg/g FW; and total flavonoids of 25.9, 24.9 and 20.3 mg catechin/100 g FW, respectively. The genetics of the rootstock and scion have been shown to be the most important factors affecting the enzyme activities and content of phenolic compounds.

The published content of rutin in leaves ranged from 3.50 to 3.99 mg/100 g FW for cultivars, and from 2.99 to 5.06 mg/100 g FW for rootstocks; chlorogenic acid ranged from 18.37 to 34.00 mg/100 g FW for cultivars, and from 23.12 to 25.07 mg/100 g FW for rootstocks. Unlike these results published by Parvaneh et al., the content of pharmacologically valuable rutin in the leaves of all cultivars tested in our study was always substantially higher than the contents of chlorogenic acid, and the reasons for this phenomenon deserve more detailed attention (the effects of the cultivar, location, seasonal variability, or the possible loss of chlorogenic acid during the drying process or storage) [38].

From the data explored in the present study, it is evident that the bark is the most important source of phloridzin (up to 91.7 mg g^−1^ DW), while rutin and phloretin are contained mainly in the leaves (up to 10.5 mg g^−1^ DW and 2.8 mg g^−1^ DW, resp.). High levels of chlorogenic acid are characteristic for flower buds (up to 3.3 mg g^−1^ DW).

### 2.2. Content of Phloridzin and Chlorogenic Acid in Whole Apple Fruits

For the completeness of the study, the contents of phloridzin and related compounds in the whole apple fruits of the four investigated cultivars were evaluated (Supplementary Materials, Section 2, Table S6). Because of the lack of fruits from individual trees, the fruits from five examined trees of each cultivar were collected together and analysed as one average sample. The contents of phloridzin in the whole fruits ranged from 52 µg g^−1^ FW (cv. ‘Rozela’) to 62 µg g^−1^ FW (cv. ‘Jonagold’); phloretin and rutin were not detected. The content of chlorogenic acid ranged from 77 µg g^−1^ FW in cv. ‘Opal’ to 424 µg g^−1^ FW in cv. ‘Rozela’.

In recent years, several systematic studies exploring the content of phloridzin in apple fruits of miscellaneous origin have been published. The highly extensive comparative assessment of 140 cultivated and wild accessions of apple trees in China was accomplished [25]. Of these, 80 were cultivars (group C), including 78 from *M. domestica* and two from *M. pumila*; the remaining 60 were wild relatives (group W), including 16 from *M. sieversii* (group Sie). The average content of phloridzin in the above specified groups was 60 µg g^−1^ FW (group C), 245 µg g^−1^ FW (group W) and 198 µg g^−1^ FW (group Sie) in apple fruit peel, and 7.3 µg g^−1^ FW, 14.7 µg g^−1^ FW and 16.0 µg g^−1^ FW in the flesh, respectively. Thus, the wild accessions demonstrated much higher contents of phloridzin in their fruits than the cultivars, especially in their peel. For comparison, the average content of phloridzin in whole apple fruits according to the Phenol-Explorer Database is 26.9 µg g^−1^ FW [52]. Thus, the phloridzin content in whole apple fruits assessed in our study was approximately twice as high as the average value reported by Neveu et al. [52].

The fruits of the midget crab apple *Malus micromalus* Makino (China) were analysed by Zhang et al. using ultrasonic-assisted aqueous two-phase extraction (35% ethanol, 16% ammonium sulfate) [53]. The average content of phloridzin in the lyophilized fruit powder was 860 µg g^−1^ DW. The distribution of phenolic compounds between peels and whole apple fruits was investigated by Lata et al., who performed an analysis of 19 apple cultivars (Poland) [54]. Separated apple tissues were ground to a fine powder in liquid nitrogen and extracted with pure methanol. The content of phloridzin ranged from 710 to 242 µg g^−1^ DW in the peel (average value 155 µg g^−1^ DW), and from 110 to 430 µg g^−1^ DW in whole apple fruits (average value 270 µg g^−1^ DW, after discarding the cores and seeds). Considering the average water content in apple fruits of about 85%, the values published by Lata et al. for whole apple fruits converted to fresh weight ranged from 16.5 to 64.5 µg g^−1^ FW, and they are comparable with the values established in the present study [54].

Generally, the highest content of phloridzin can be found in separated apple seeds. Xu et al. analysed the content of phloridzin in the seeds of seven cultivars grown in China; the results ranged from 2405 to 8644 µg g^−1^ DW [12].

In recent years, great attention has also been devoted to the research of storage conditions and their influence on the bioactive compounds in apple fruits. Ma et al. investigated the influence of storage time, temperature and 1-methylcyclopropene (1-MCP) treatment on the content of phenolic compounds in apple peel and flesh tissues (cv. ‘Jonagold’, China) [13]. The content of phloridzin after 180 days of storage was 38.0 µg g^−1^ FW in the peel and 4.0 µg g^−1^ FW in the flesh. The influence of the storage time and 1-MCP treatment was not too significant, especially according to the changes in the phloridzin content. Chen et al. investigated the influence of fruit bagging on the content of phenolic compounds in the apple fruits of three cultivars grown in China (‘Golden Delicious’, ‘Red Delicious’ and ‘Royal Gala’) [55]. The reported results revealed the relatively low impact of bagging on the phloridzin content, and considerable differences among cultivars (approx. 15.0–45.0 µg g^−1^ FW in the flesh of unbagged fruits).

In another study, Bílková et al. assessed the amount of phloridzin, chlorogenic acid, rutin, epicatechin and quercitrin in the apples of ten cultivars after their harvest and storage under ultra-low oxygen conditions [56]. The phloridzin content immediately after the harvest ranged from 0.7 µg g^−1^ FW in ‘Rubín’ to 13.6 µg g^−1^ FW in ‘Angold’; the chlorogenic acid content ranged from 1.4 µg g^−1^ FW in ‘Fragrance’ to 99.6 µg g^−1^ FW in ‘Angold’; and the rutin content ranged from 0.5 µg g^−1^ FW in ‘Golden Delicious’ to 16.8 µg g^−1^ FW in ‘UEB 32 642’. The reported results proved the benefits of long-term ultra-low oxygen storage in contrast to short-term storage.

The data obtained in our study showed the substantially higher content of phloridzin in all four tested cultivars. Similarly, in the study of Bílková et al., no phloretin in fresh fruits was detected [56]. Regarding the chlorogenic acid content, cv. ‘Jonagold’ is comparable to cv. ‘Angold’ (140 vs. 99.6 µg g^−1^ FW), cv. ‘Opal’ is comparable to cv. ‘Gala’ (77 vs. 52.5 µg g^−1^ FW), and cv. ‘Redlane’ and ‘Rozela’ contained much higher levels of chlorogenic acid (330 and 424 µg g^−1^ FW).

### 2.3. Statistical Comparison of the Analysed Cultivars

The highest concentration of phloridzin was found in the bark. In March, the *p*-value of the ANOVA test was *p* = 0.004, i.e., the average concentration across the cultivars was not the same, with a test error of 0.4%. According to the Scheffe’s test, only cv. ‘Rozela’ differed significantly from other cultivars. In May, the *p*-value of the ANOVA test was *p* = 0.001, i.e., the average concentration across the cultivars was not the same, with a test error 0.1%. According to Scheffe’s test, cv. ‘Jonagold’ did not differ from cv. ‘Opal’, and similarly cv. ‘Rozela’ did not differ from cv. ‘Redlane’. However, the differences between these pairs were significant. In July, the *p*-value of the ANOVA test was *p* = 0.114, i.e., the average concentration across the cultivars was not the same, with a test error 11.4%. Only cv. ‘Rozela’ differed from the others. In September, the *p*-value of the ANOVA test was *p* = 0.027, i.e., the average concentration across the cultivars was not the same, with a test error of 2.7%. Only cv. ‘Redlane’ differed from the other cultivars.

The content of phloridzin varied considerably according to the type of matrix and the vegetation period. In the case of bark, significant seasonal variability was proved for all of the tested cultivars, with a maximum in May. In the case of leaves, the results varied significantly during the vegetation period, with a considerable upward trend from May to September (except for cv. ‘Jonagold’). In the case of twigs, the seasonal variations were less distinctive. After basic statistical evaluation, we found a negative correlation between the content of phloridzin in the bark and leaves (r^2^ = −0.6543) during the vegetation period from May to September. A significant decreasing trend of phloridzin concentrations in the bark is followed by an upward trend of phloridzin in the leaves. The data are presented in the Supplementary Materials section in Graph S1.

Phloretin was found only in the leaves, with a maximum of 2.79 mg g^−1^ DW in May (cv. ‘Jonagold’). The phloretin levels did not differ significantly between the cultivars, and no clear trends in seasonal variability could be found. The levels of chlorogenic acid were rather low to zero. The only exceptions were the buds (highest value 3.29 mg g^−1^ DW) and bark in March. The cultivar did not play a significant role in the concentration of chlorogenic acid. Rutin was concentrated mainly in the leaves (the highest value was 10.50 mg g^−1^ in September) and in the bark. Higher levels of rutin were found in samples from the later months of the growing season, i.e., in July and September. The concentration of rutin in the individual cultivars varied according to the month and matrix. For the leaves, the ‘Redlane’ and ‘Opal’ cultivars proved to be more profitable; for the other matrices, the effect of the cultivar was not significant.

## 3. Materials and Methods

### 3.1. Plant Material

Four apple tree cultivars (*Malus domestica*)—‘Jonagold’, ‘Opal’, ‘Redlane’ and ‘Rozela’—were investigated in this study. All of the trees were cultivated at the Experimental Orchard of the Department of Horticulture (50°7′22.486″ N, 14°23′58.181″ E, altitude 196 m), Czech University of Life Sciences in Prague, Czech Republic.

The cultivars ‘Jonagold’ and ‘Opal’, both on the rootstock M9, and ‘Rozela’ on the rootstock M7, age 4 years, were planted in spring 2014 into a 4.0 × 1.5 m space and were shaped as a slender spindle. The cv. ‘Redlane’ on the rootstock M26, age 5 years, was columnar in shape, and was planted in spring 2013 into a 3.0 × 0.8 m space. The whole orchard was under drip irrigation.

The cv. ‘Jonagold’ (‘Golden Delicious’ × ‘Jonathan’) was bred in the USA and has become famous throughout the world. The cultivars ‘Opal’ (‘Golden Delicious’ × ‘Topaz’), ‘Rozela’ (‘Vanda’ × ‘Bohemia’) and ‘Redlane’ (‘Maypole’ × UEB 2345/1) were bred in the Czech Republic, Institute of Experimental Botany of the Czech Academy of Sciences in Prague. All these three cultivars had monogenic resistance against apple scab (*Venturia floribunda*, Vf).

The samples were always taken from five selected trees of each cultivar in four-time terms during the vegetation period of 2018 (27 March, 31 May, 31 July, and 27 September). The analysed parts of the apple trees were young shoots (twigs) about 20 cm long (in all four terms), samples of bark from twigs (all four terms), flower buds (only in March) and leaves (all terms excluding March). At the last term in September, the samples of the fruits from each cultivar were taken and collected. All samples were stored in a refrigerator at about 4 °C until the next processing.

### 3.2. Preparation and Extraction of the Samples

All samples (except for the fruits) were desiccated in a drying chamber at 55 °C for 24 h. After that, the samples were pulverised into a fine powder on a laboratory mill (Fritsch Pulverisette 14, Fritsch GmbH, Idar Oberstein, Germany). In total, 100 mg of each pulverised sample was weighed into a centrifugation test tube, and 2 mL of the extraction agent was added (a mixture of 70 mL absolute ethanol, 30 mL deionized water and 2 mL formic acid); the test tubes were closed using parafilm. The absolute ethanol GR grade was purchased from Lachner (Neratovice, Czech Republic), and the formic acid reagent (≥95%) was purchased from Merck Sigma-Aldrich (St. Louis, MO, USA; KGaA, Darmstadt, Germany). Adjusted deionized ultra-pure water was prepared using Simplicity UV (Merck Millipore, KGaA, Darmstadt, Germany). The samples were vortexed at 1800 rpm for 2 min (Basic 3, IKA Werke GmbH & Co. KG, Staufen, Germany), and then centrifuged at 5000 rpm (3186 rcf) for 10 min (5810R, Eppendorf, Hamburg, Germany). The supernatant was removed by means of a glass pipette equipped with a balloon and transferred into a 10 mL volumetric flask. The extraction of each sample was repeated twice using 1.5 mL extraction agent, and all portions were collected in the volumetric flask, which was filled up to the mark using the extraction agent and shaken thoroughly. Then, approximately 2 mL of each extract were taken with a plastic syringe and filtered through a 0.45 µm Durapore^®^ polyvinylidene fluoride disc filter (PVDF, Merck KGaA, Darmstadt, Germany) into a 1.5 mL dark brown vial.

### 3.3. Chromatography Analysis and the Evaluation of the Results

Commercially available standards of phloridzin (≥99%), phloretin (≥99%), chlorogenic acid (≥95%), rutin (≥94%), HPLC gradient grade acetonitrile and phosphoric acid were purchased from Merck Sigma-Aldrich (St. Louis, MO, USA, Merck KGaA, Darmstadt, Germany).

A Shimadzu LC-10AD (Shimadzu Corporation, Kyoto, Japan) HPLC system equipped with an LC-10AD binary solvent delivery module, with an SIL-HTA autosampler, a DGU-14A on-line degasser, an SPD-M10A diode array detector, and a CTO-10AC column oven (Shimadzu Co, Kyoto, Japan) was used in this study. The data evaluation and data acquisition were performed using the Shimadzu “LC Lab-Solution” software (Shimadzu Corporation, Kyoto, Japan).

The separation was performed on an YMC-Triart C18 ExRS (150 × 4.6 mm) chromatography column (YMS Co, Ltd., Japan, Kyoto) with a particle size of 5 µm (pore size 8 nm) at a 30 °C temperature. The mobile phase consisted of a water solution of 0.1% phosphoric acid (A) and acetonitrile (B) at a flow rate 1.0 mL/min according to the following elution gradient program: 0.01–10 min 10% mobile phase B, 10–10.2 min 50% mobile phase B, 10.2–12.5 min 10% mobile phase B. An injection volume of 1 µL was used. The peaks of separated compounds were detected at the wavelength of 280 nm for phloridzin and phloretin, 327 nm for chlorogenic acid, and 354 nm for rutin. All analysed compounds were successfully separated within 13 min.

The identification of the peaks was achieved by comparing their retention times and UV spectra with reference standards. The quantitative evaluation of the peaks was calculated using the method of the external standard:

$$c_{x}=\frac{A_{x}}{A_{ST}}*c_{ST}$$

where *c_x_* is the concentration of the analyte in a sample (mg L)^−1^, *c_ST_* is the concentration of the corresponding standard (mg L)^−1^, *A_x_* is the peak area of the analyte in a sample, and *A_ST_* is the peak area of the standard.

The concentration of a determined compound was converted to the amount of extraction solvent used, followed by the conversion to the real sample weight with the correction to the purity of the used standard. The total amount of individual phenolic compounds was expressed in mg g^−1^ of dried plant material, or in µg g^−1^ of fresh fruit material. The basic validation parameters of the chromatography method and the chromatographic patterns of real extracts are shown in the Supplementary Materials, Section 1.

## 4. Conclusions

Dihydrochalcones are important antidiabetics, sweeteners and antioxidants that provide benefits to human health. The results of this study provide new knowledge about the composition and contents of phenolic compounds of four apple tree cultivars, which were assessed for the main dihydrochalcones, phloridzin and phloretin, phenolic chlorogenic acid and flavonoid rutin levels in their individual parts, and their changes during the vegetation period of March–September. Their levels depend mainly on the part of tree (bark, twigs, leaves, flower buds, fruits), but also on the apple tree cultivar and on the month of vegetation period. In the present study, apple tree bark, leaves, and twigs were proven to be important sources of phloridzin, containing up to 9.17%, 8.25% (cv. ‘Opal’) and 5.24% (cv. ‘Rozela’) of this valuable bioactive compound in their dried mass, respectively. Apple leaves were found to be an important source of rutin (up to 10.5% in the dried mass, cv. ‘Opal’). In conclusion, the tested plant material covering the tree bark, twigs, and leaves was found to be important source of bioactive plant metabolites, especially phloridzin and rutin. Therefore, the simple availability of waste plant material can be used as a rich source of phenolic compounds for cosmetics, nutraceuticals, and food supplement preparation.

## Data Availability

Not applicable.

## Supplementary Materials

The following are available online. Figure S1: Calibration curve of chlorogenic acid, Figure S2: Calibration curve of rutin, Figure S3: Calibration curve of phloridzin, Figure S4: Calibration curve of phloretin, Figure S5: Chromatogram of phenolic compounds from apple leaf extract of ‘Opal’ cultivar, Figure S6: Chromatogram of phenolic compounds from apple bark extract of ‘Opal’ cultivar, Figure S7: Chromatogram of phenolic compounds from apple twig extract of ‘Opal’ cultivar, Figure S8: Chromatogram of phenolic compounds from apple bud extract of ‘Opal’ cultivar, Table S1: Basic validation parameters, Table S2: Content of phloridzin and related compounds in cv. ‘Jonagold’ during vegetation period, Table S3: Content of phloridzin and related compounds in cv. ‘Opal’ during vegetation period, Table S4: Content of phloridzin and related compounds in cv. ‘Redlane’ during vegetation period, Table S5: Content of phloridzin and related compounds in cv. ‘Rozela’ during vegetation period, Table S6: Content of phloridzin and related compounds in whole apple fruits, Graph S1: Relationship between the content of phloridzin (mg g^−1^ of DW) in bark and leaves during the vegetation period from May to September for all tested cultivars.

## Sample Availability

Samples of the compounds phloridzin, phloretin, chlorogenic acid, rutin, and plant extracts are available from the authors.
