# Supplementary material for: Dynamics of Phloridzin and Related Compounds in Four Cultivars of Apple Trees during the Vegetation Period
Source: Molecules. 2021 Jun 22;26(13):3816. doi: 10.3390/molecules26133816 (PMC8270342; doi:10.3390/molecules26133816)

Dynamics of Phloridzin and Related Compounds in Four Cultivars of Apple Trees During the Vegetation Period

Jan Táborský [1], Josef Sus [2], Jaromír Lachman [1], Barbora Šebková [1], Anežka Adamcová [3] and Dalibor Šatínský [3,*]

[1] Department of Chemistry, Faculty of Agrobiology, Food and Natural Resources, Czech University of Life Sciences in Prague, Czech Republic
[2] Department of Horticulture, Faculty of Agrobiology, Food and Natural Resources, Czech University of Life Sciences in Prague, Czech Republic
[3] Department of Analytical Chemistry, Faculty of Pharmacy in Hradec Králové, Charles University, Czech Republic
* Corresponding author: satinsky@faf.cuni.cz, Tel.: +42-049-506-7228

Supplementary Materials

1. HPLC Method 2
1.1. Basic Validation Parameters 2
1.2. Calibration Curves of Phenolic Compounds 3
1.3. Chromatograms of Real Extracts 4
2. Content of Phloridzin and Other Compounds in 4 Cultivars During the Vegetation Period 6

1. HPLC Method

1.1. Basic validation parameters

Table S1. Basic validation parameters.

**Analyte**	**t_R[a] (min)**	r[b]	**Calibration Range (mg L^{-1})**	**LOD[c] (mg L^{-1})**	**LOQ[d] (mg L^{-1})**
Chlorogenic acid	4.48	0.997	2–250	0.078	0.260
Rutin	6.29	0.998	2–250	0.145	0.434
Phloridzin	7.55	0.994	1000–8000	0.080	0.263
Phloretin	10.52	0.998	2–250	0.098	0.324

[a] Retention time. [b] Correlation coefficient. [c] Limit of detection. [d] Limit of quantitation.

1.2. Calibration Curves of Phenolic Compounds

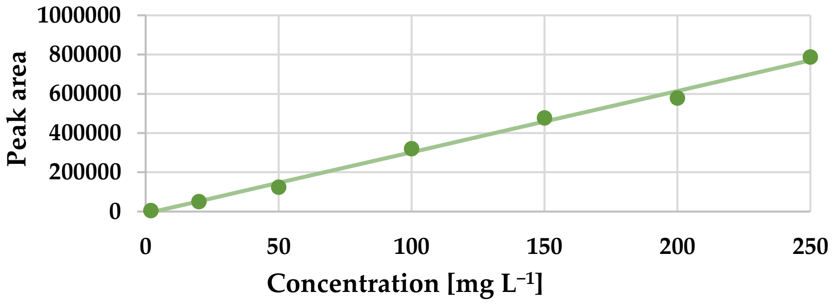

Figure S1. Calibration curve of chlorogenic acid.

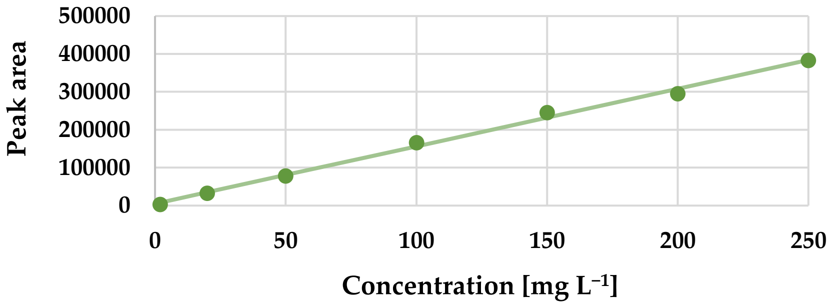

Figure S2. Calibration curve of rutin.

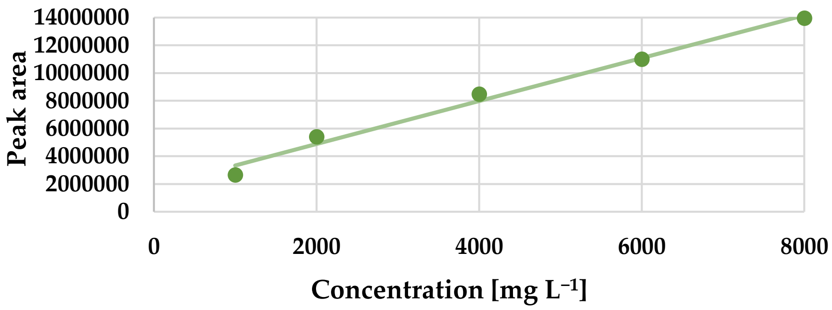

Figure S3. Calibration curve of phloridzin.

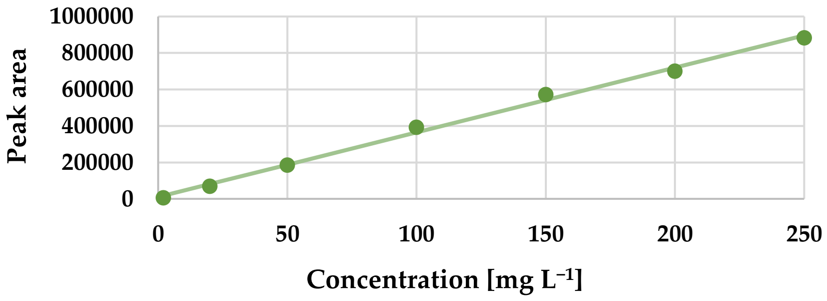

Figure S4. Calibration curve of phloretin.

1.3. Chromatograms of Real Extracts

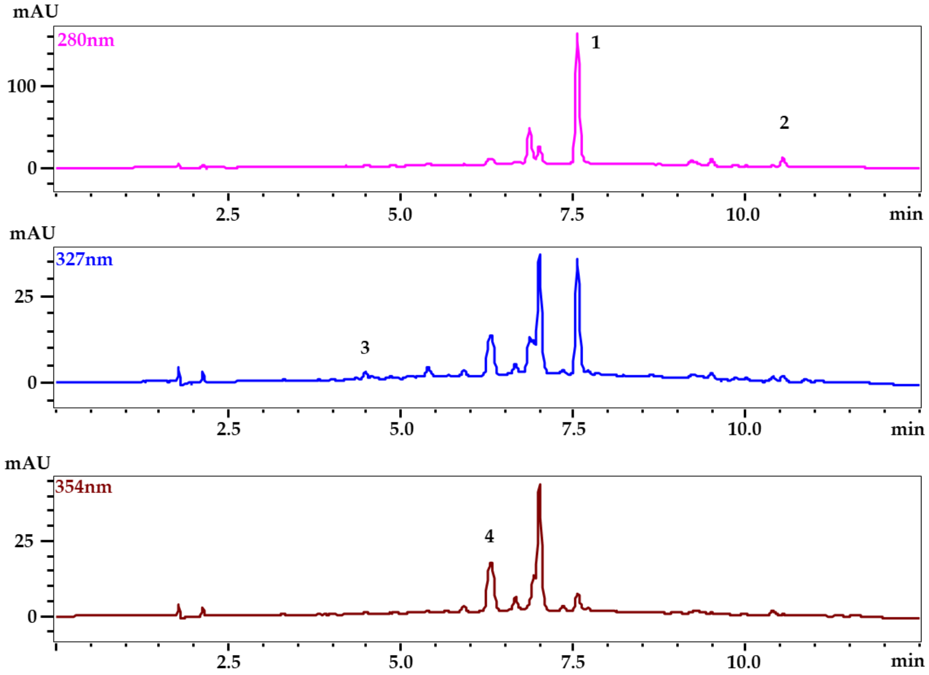

Figure S5. Chromatogram of phenolic compounds from apple leaf extract of ´Opal´ cultivar. 1—phloridzin, 2—phloretin, 3—chlorogenic acid, 4—rutin.

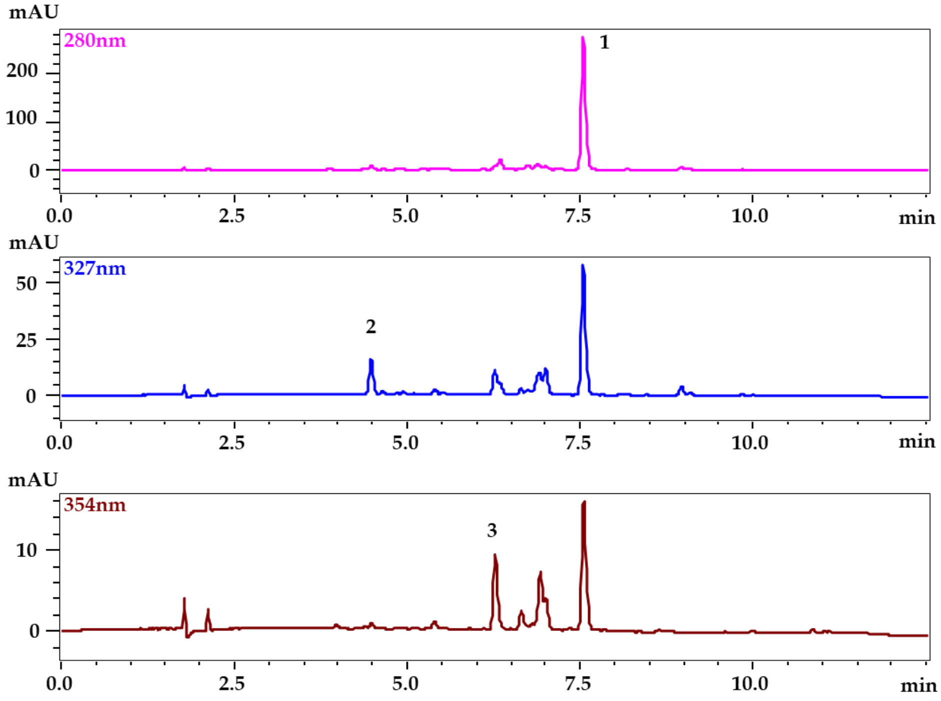

Figure S6. Chromatogram of phenolic compounds from apple bark extract of ´Opal´ cultivar. 1—phloridzin, 2—chlorogenic acid, 3—rutin.

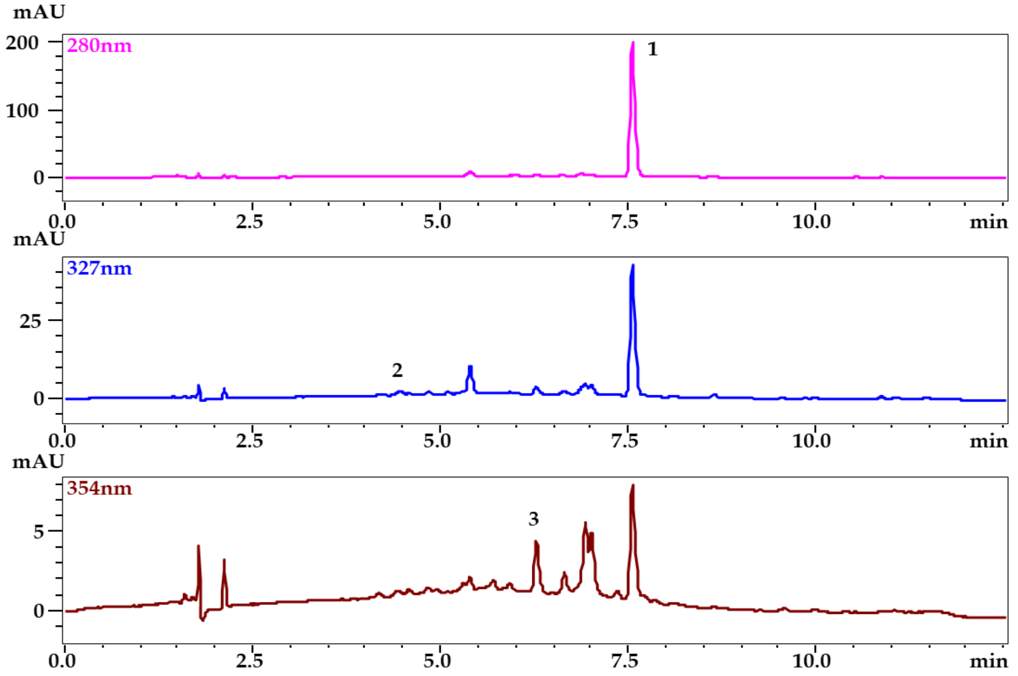

Figure S7. Chromatogram of phenolic compounds from apple twig extract of ´Opal´ cultivar. 1—phloridzin, 2—chlorogenic acid, 3—rutin.

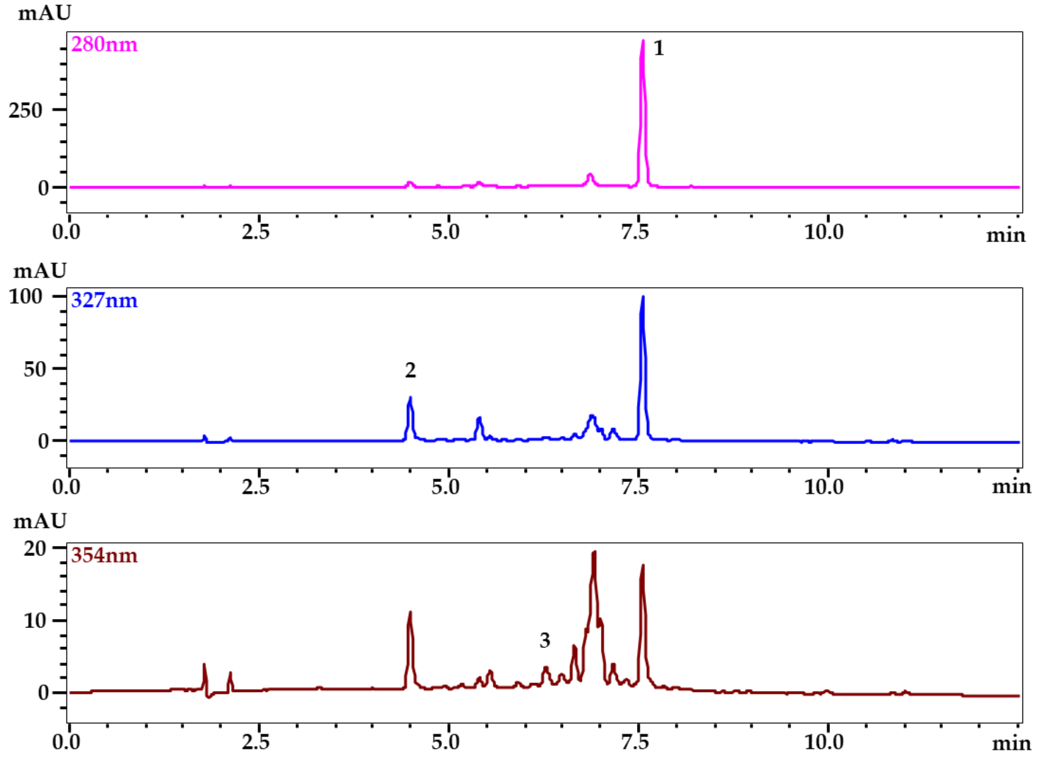

Figure S8. Chromatogram of phenolic compounds from apple bud extract of ´Opal´ cultivar. 1—phloridzin, 2—chlorogenic acid, 3—rutin.

2. Content of Phloridzin and Other Compounds in 4 Cultivars During the Vegetation Period

Table S2: Content of phloridzin and related compounds in cv. 'Jonagold' during vegetation period (all values in mg g^{-1} of dried weight (DW), SD = standard deviation, n = 5).

Part of Tree	Compound	March	May	July	September
		mg g^{-1} ± SD	mg g^{-1} ± SD	mg g^{-1} ± SD	mg g^{-1} ± SD
Twigs	Phloridzin	12.85 ± 1.95	33.77 ± 4.23	30.47 ± 6.13	28.74 ± 5.40
	Phloretin	0.00 ± 0.00	0.00 ± 0.00	0.00 ± 0.00	0.00 ± 0.00
	Chlorog. acid	0.21 ± 0.06	0.00 ± 0.00	0.19 ± 0.07	0.32 ± 0.08
	Rutin	0.28 ± 0.05	0.76 ± 0.18	0.41 ± 0.11	0.94 ± 0.21
Bark	Phloridzin	57.08 ± 7.60	90.47 ± 3.43	73.33 ± 11.80	29.26 ± 8.25
	Phloretin	0.00 ± 0.00	0.00 ± 0.00	0.00 ± 0.00	0.00 ± 0.00
	Chlorog. acid	1.03 ± 0.32	0.00 ± 0.00	0.00 ± 0.00	0.00 ± 0.00
	Rutin	0.00 ± 0.00	3.00 ± 0.73	2.49 ± 0.45	1.51 ± 0.54
Flower buds (March) or leaves (May-September)	Phloridzin	34.64 ± 14.02	25.50 ± 13.91	49.71 ± 9.63	34.92 ± 9.12
	Phloretin	0.00 ± 0.00	2.79 ± 1.43	1.06 ± 0.57	0.41 ± 0.11
	Chlorog. acid	2.59 ± 1.60	0.16 ± 0.03	0.19 ± 0.02	0.24 ± 0.08
	Rutin	0.00 ± 0.00	3.09 ± 0.66	7.29 ± 0.26	5.22 ± 1.56

Table S3. Content of phloridzin and related compounds in cv. 'Opal' during vegetation period (all values in mg g^{-1} of dried weight (DW), SD = standard deviation, *n* = 5).

Part of Tree	Compound	March	May	July	September
		mg g^{-1} ± SD	mg g^{-1} ± SD	mg g^{-1} ± SD	mg g^{-1} ± SD
Twigs	Phloridzin	13.27 ± 0.81	28.14 ± 2.95	45.97 ± 8.43	33.54 ± 4.47
	Phloretin	0.00 ± 0.00	0.00 0.00	0.00 ± 0.00	0.00 ± 0.00
	Chlorog. acid	0.20 ± 0.03	0.00 ± 0.00	0.31 ± 0.12	0.50 ± 0.10
	Rutin	0.29 ± 0.03	0.64 ± 0.12	0.65 ± 0.19	0.99 ± 0.16
Bark	Phloridzin	57.42 ± 5.90	91.74 ± 4.42	73.56 ± 21.67	25.93 ± 9.85
	Phloretin	0.00 ± 0.00	0.00 ± 0.00	0.00 ± 0.00	0.00 ± 0.00
	Chlorog. acid	1.17 ± 0.19	0.00 ± 0.00	0.27 ± 0.13	0.15 ± 0.07
	Rutin	3.50 ± 0.65	4.11 ± 0.97	2.69 ± 0.89	0.76 ± 0.28
Flower buds (March) or leaves (May-September)	Phloridzin	53.94 ± 14.76	31.33 ± 6.39	47.14 ± 8.63	82.47 ± 21.96
	Phloretin	0.00 ± 0.00	1.98 ± 0.64	1.21 ± 0.21	1.08 ± 0.47
	Chlorog. acid	2.86 ± 0.59	0.22 ± 0.04	0.12 ± 0.03	0.53 ± 0.07
	Rutin	0.52 ± 0.08	5.23 ± 0.86	7.82 ± 2.04	10.50 ± 2.93

Table S4. Content of phloridzin and related compounds in cv. 'Redlane' during vegetation period (all values in mg g^{-1} of dried weight (DW), SD = standard deviation, *n* = 5).

Part of Tree	Compound	March	May	July	September
		mg g^{-1} ± SD	mg g^{-1} ± SD	mg g^{-1} ± SD	mg g^{-1} ± SD
Twigs	Phloridzin	12.94 ± 2.39	36.27 ± 4.69	39.41 ± 4.99	29.30 ± 1.42
	Phloretin	0.00 ± 0.00	0.00 ± 0.00	0.00 ± 0.00	0.00 ± 0.00
	Chlorog. acid	0.14 ± 0.09	0.00 ± 0.00	0.18 ± 0.02	0.74 ± 0.08
	Rutin	0.61 ± 0.16	0.33 ± 0.12	0.66 ± 0.10	1.08 ± 0.16
Bark	Phloridzin	53.74 ± 7.85	77.12 ± 6.91	74.97 ± 9.56	43.14 ± 5.67
	Phloretin	0.00 ± 0.00	0.00 ± 0.00	0.00 ± 0.00	0.00 ± 0.00
	Chlorog. acid	1.32 ± 0.48	0.00 ± 0.00	0.00 ± 0.00	0.95 ± 0.46
	Rutin	3.58 ± 0.82	1.51 ± 0.41	2.36 ± 0.71	1.74 ± 0.35
Flower buds (March) or leaves (May-September)	Phloridzin	46.12 ± 4.46	10.96 ± 3.14	36.17 ± 6.94	39.69 ± 3.61
	Phloretin	0.00 ± 0.00	0.91 ± 0.52	1.32 ± 0.42	0.89 ± 0.20
	Chlorog. acid	2.63 ± 0.44	0.00 ± 0.00	0.26 ± 0.07	0.75 ± 0.22
	Rutin	1.27 ± 0.23	2.17 ± 0.35	8.83 ± 2.27	9.18 ± 2.03

Table S5. Content of phloridzin and related compounds in cv. 'Rozela' during vegetation period (all values in mg g^{-1} of dried weight (DW), SD = standard deviation, *n* = 5).

Part of Tree	Compound	March	May	July	September
		mg g^{-1} ± SD	mg g^{-1} ± SD	mg g^{-1} ± SD	mg g^{-1} ± SD
Twigs	Phloridzin	13.92 ± 1.96	32.40 ± 9.84	28.17 ± 3.13	52.37 ± 12.07
	Phloretin	0.00 ± 0.00	0.00 ± 0.00	0.00 ± 0.00	0.00 ± 0.00
	Chlorog. acid	0.23 ± 0.08	0.00 ± 0.00	0.19 ± 0.09	0.55 ± 0.16
	Rutin	0.41 ± 0.13	0.47 ± 0.07	0.57 ± 0.12	1.52 ± 0.56
Bark	Phloridzin	41.06 ± 5.57	77.61 ± 6.81	55.25 ± 12.13	31.53 ± 9.15
	Phloretin	0.00 ± 0.00	0.00 ± 0.00	0.00 ± 0.00	0.00 ± 0.00
	Chlorog. acid	1.40 ± 0.21	0.00 ± 0.00	0.00 ± 0.00	0.12 ± 0.03
	Rutin	3.58 ± 0.94	2.52 ± 0.86	1.91 ± 0.71	1.67 ± 0.74
Flower buds (March) or leaves (May-September)	Phloridzin	49.39 ± 5.25	18.80 ± 2.78	32.60 ± 8.31	66.48 ± 26.28
	Phloretin	0.00 ± 0.00	1.06 ± 0.31	0.49 ± 0.13	1.06 ± 0.40
	Chlorog. acid	3.29 ± 1.03	0.00 ± 0.00	0.00 ± 0.00	0.58 ± 0.22
	Rutin	1.26 ± 0.29	3.03 ± 0.68	5.09 ± 2.34	8.54 ± 2.05

Table S6. Content of phloridzin and related compounds in whole apple fruits (μg g^{-1} FW), standard deviation, *n* = 1).

Compound	´Jonagold´	´Opal´	´Redlane´	´Rozela´
Phloridzin	62	54	58	52
Phloretin	nd	nd	nd	nd
Chlorogenic acid	140	77	330	424
Rutin	nd	nd	nd	nd

** Samples of apple cultivars were prepared by homogenization of 5 whole fruits. nd—not detected.*

Graph S1. Relationship between the content of phloridzin (mg g^{-1} of DW) in bark and leaves during the vegetation period from May to September for all tested cultivars.

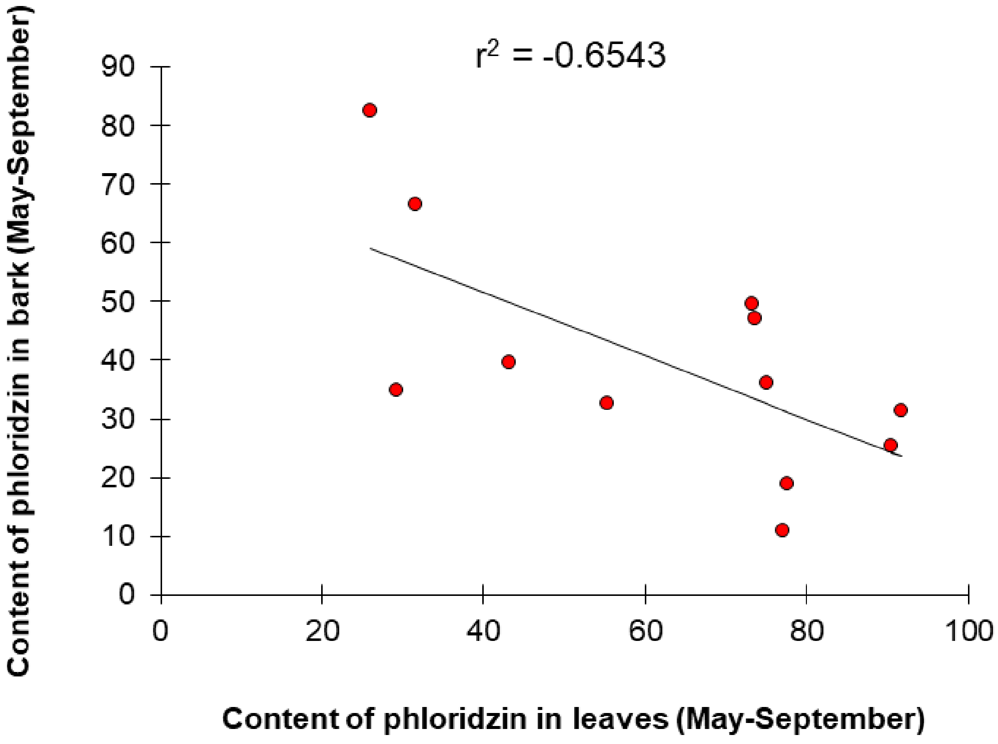

Supplement: Supplementary file 1 [file molecules-26-03816-s001.zip › molecules-1229143-supplementary.pdf]
